# The value of quantitative analysis of radionuclide bone SPECT/CT imaging in vertebral compression fracture: a retrospective study

**DOI:** 10.1186/s12880-024-01452-9

**Published:** 2024-10-08

**Authors:** Yuhua Wang, Feifei Qiao, Na Li, Ye Liu, Yahong Long, Kang Xu, Jiantao Wang, Wanchun Zhang

**Affiliations:** 1 Department of Nuclear Medicine, Shanxi Bethune Hospital, Shanxi Academy of Medical Sciences, Third Hospital of Shanxi Medical University, Tongji Shanxi Hospital, Long Cheng Street 99, Xiao Dian District, Taiyuan, 030032 China; 2 Department of Head and Neck Surgery, Shanxi Cancer Hospital, Zhigongxincun No.3, Xinghualing District, Taiyuan, 030013 China

**Keywords:** Vertebral compression fractures, Quantitative technique, Bone SPECT/CT, Prediction

## Abstract

**Background:**

Most patients with osteoporosis experience vertebral compression fracture (VCF), which significantly reduces their quality of life. These patients are at a high risk of secondary VCF regardless of treatment. Thus, accurate diagnosis of VCF is important for treating and preventing new fractures. We aimed to investigate the diagnostic and predictive value of quantitative bone imaging techniques for fresh VCF.

**Methods:**

From November 2021 to March 2023, 34 patients with VCF were enrolled in this study, all of whom underwent routine ^99m^Tc-MDP whole-body bone planar scan and local SPECT/CT imaging. The maximum standard uptake value (SUVmax) of 57 fresh VCF, 57 normal adjacent vertebrae, and 19 old VCF were measured. Based on the site of the fracture, fresh VCFs were regrouped into the intervertebral-type group and the margin-type group. Meanwhile, 52 patients who had no bone metastasis or VCFs in their bone scan were assigned to the control group. The SUVmax of 110 normal vertebral bodies and 10 old VCFs in the control group were measured.

**Results:**

The median SUVmax of fresh VCF was 19.80, which was significantly higher than the SUVmax of other groups. The receiver operator characteristic (ROC) curve showed that the cut-off value of SUVmax was 9.925 for diagnosing fresh VCF. The SUVmax in the intervertebral-type group was significantly higher than that in the margin-type group (*P* = 0.04). The SUVmax of normal vertebrae was higher among patients than among the control group (*P*<0.01), but the CT HU value showed no significant difference.

**Conclusion:**

The quantitative technique of bone SPECT/CT has a significant value in diagnosing fresh VCF. It can also determine the severity of fractures. In addition, whether the SUVs of the vertebrae adjacent to the fractured vertebra can predict re-fracture deserves further studies.

## Background

The population is aging as people’s life expectancy increases, and senile osteoporosis has become a major global health problem. Vertebral compression fracture (VCF) is a frequent and unpredictable complication of osteoporosis [1]. Approximately 25% of patients with osteoporosis experience VCF which significantly reduces their quality of life due to pain and disability [2], and patients are at high risk of secondary VCF regardless of treatment [3]. Thus, accurate diagnosis of VCF is important for treating and preventing new fractures [1]. However, acute VCF can affect multiple vertebrae, the trauma history is difficult to assess, and the symptoms may be non-specific. Therefore, it is crucial to determine the presence or absence of a fresh VCF. At this time, imaging examinations are needed to identify the affected vertebra.

Different imaging techniques have different values in diagnosing VCFs [4]. Conventional spine radiographs are non-specific for differentiating fresh VCFs from old VCFs. In addition, there is no morphological change, which is inconsistent with symptoms and makes clinical judgment difficult. Computed tomography (CT) scans cannot distinguish fresh VCFs from old VCFs when they exist simultaneously and may also miss multiple-level fractures. Magnetic resonance imaging (MRI) is considered to be the best method for diagnosing fresh VCFs because of the bone marrow edema after vertebral fracture [5]; however, some patients with contraindications cannot undergo MRI. Moreover, most patients with VCFs may have multiple fractures, and MRI cannot be used to fully assess them in the absence of a comprehensive scanning scope.

In fresh VCFs, factors such as abundant local blood flow, vigorous metabolic renewal, active osteoblast repair, and calcium salt deposition lead to the abnormal uptake of technetium-99m-methyldiphosphonate ([^99m^Tc]-MDP) in the fracture end [6]. Therefore, bone scintigraphy has a very high sensitivity for detecting the affected vertebrae in patients with VCFs [7–9]. In addition, one study adopted the hydroxyapatite-water decomposition technique in dual-energy CT for detecting fresh VCFs [10]. [^99m^Tc]-MDP is adsorbed on the hydroxyapatite surface during bone imaging. Whole-body bone scan combined with regional single photon emission computed tomography/computed tomography (SPECT/CT) can evaluate the extent of the fracture and determine whether the fracture is caused by a tumor [11]. The diagnosis of VCFs by bone imaging was mainly based on visual inspection in previous studies, which was not objective enough. With the application of quantitative techniques in SPECT/CT [12], the uptake of radiopharmaceuticals by bone lesions can also be quantified. Therefore, in this paper, a retrospective study was conducted to analyze the clinical value of quantitative bone imaging techniques for fresh VCF.

## Methods

### Clinical data

**Fig. 1:**
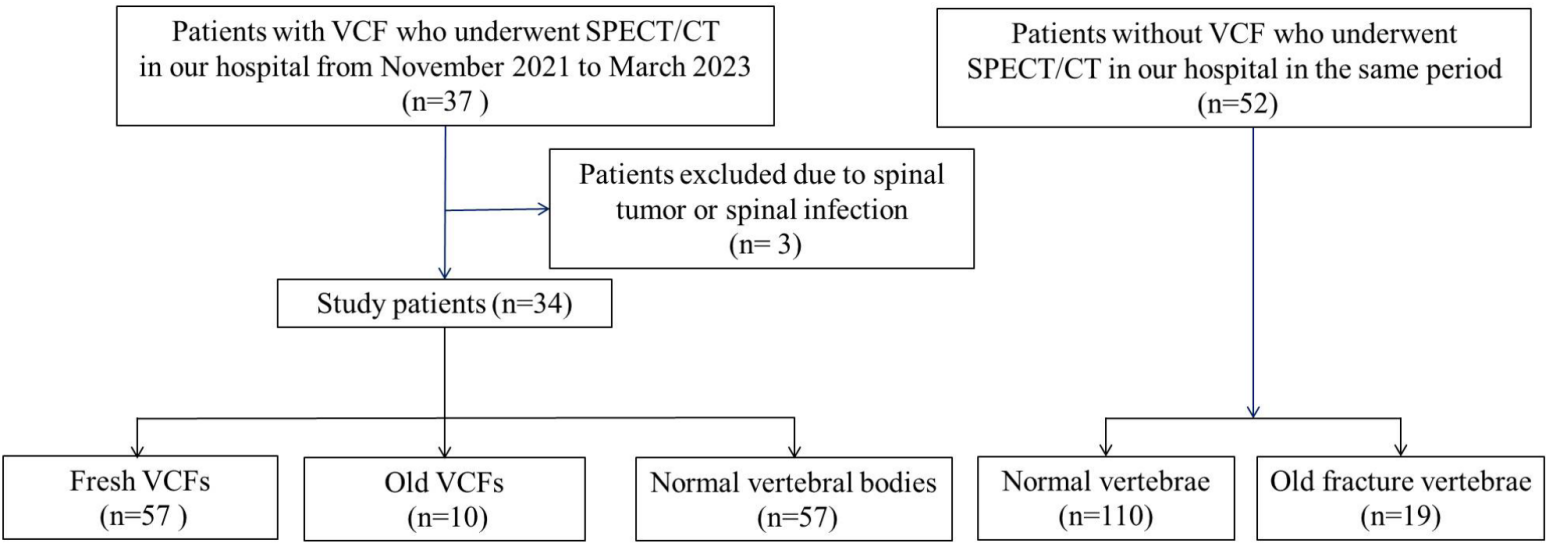
Flow diagram showing details of patient selection. VCF = vertebral compression fracture, SPECT/CT = single photon emission computed tomography/ computed tomography

This study was approved by the hospital Ethics Committee (review board number: YXLL-2023-010). We retrospectively included 34 patients diagnosed with VCF in our hospital from November 2021 to March 2023. All patients underwent bone scan before treatment. The inclusion criteria were as follows: clinical diagnosis of VCF with a recent history of trauma or low-energy injury. The exclusion criteria were as follows: fractures caused by tumors or infectious diseases. Subjects with a cohort of age 60–80 (*n* = 52) who had no bone metastases or VCFs in bone imaging in the same period were assigned to the control group (Fig. 1).

### [^99m^Tc]-MDP scintigraphy and SPECT/CT

Whole-body bone scan was performed on a double-head gamma camera equipped with low-energy high-resolution (LEHR) collimators (Siemens Symbia Intevo Bold). Three hours (h) after injecting 740-925MBq (20-25mCi) of [^99m^Tc]-MDP, whole-body planar scans were obtained. All patients were asked to drink water after injecting and urinate immediately before imaging. Planar scintigraphy was done in Siemens using a low-energy, high-resolution collimator, 512 × 512 matrix, an energy peak of 140 keV, and a window width of 20%. Regional SPECT/CT tomographic fusion imaging was conducted immediately after planar imaging. During the SPECT study, 30 stops were acquired, with 20s acquisition time per frame and a matrix of 256 × 256 pixels. CT images were acquired with the tube voltage set at 100 kV. Tube current was determined by automatic dose modulation with a reference current of 60 mA. The ordered subset conjugate gradient minimization (OSCGM) xSPECT algorithm (Siemens Healthineers) was used to perform SUV quantification with 36 iterations and one subset.

### Qualitative and quantitative analysis of [^99m^Tc]-MDP SPECT-CT

Reconstructed images were evaluated visually and quantitatively by two experienced nuclear medicine physicians blinded to the surgical results. The extent of vertebral uptake of [^99m^Tc]-MDP was evaluated on sagittal SPECT/CT imaging. Positive [^99m^Tc]-MDP SPECT/CT scans indicated a fixed linear concentration in the spinal colon on sagittal SPECT imaging. In addition, a parenchymal change was observed in the corresponding position on CT imaging.

**Fig. 2:**
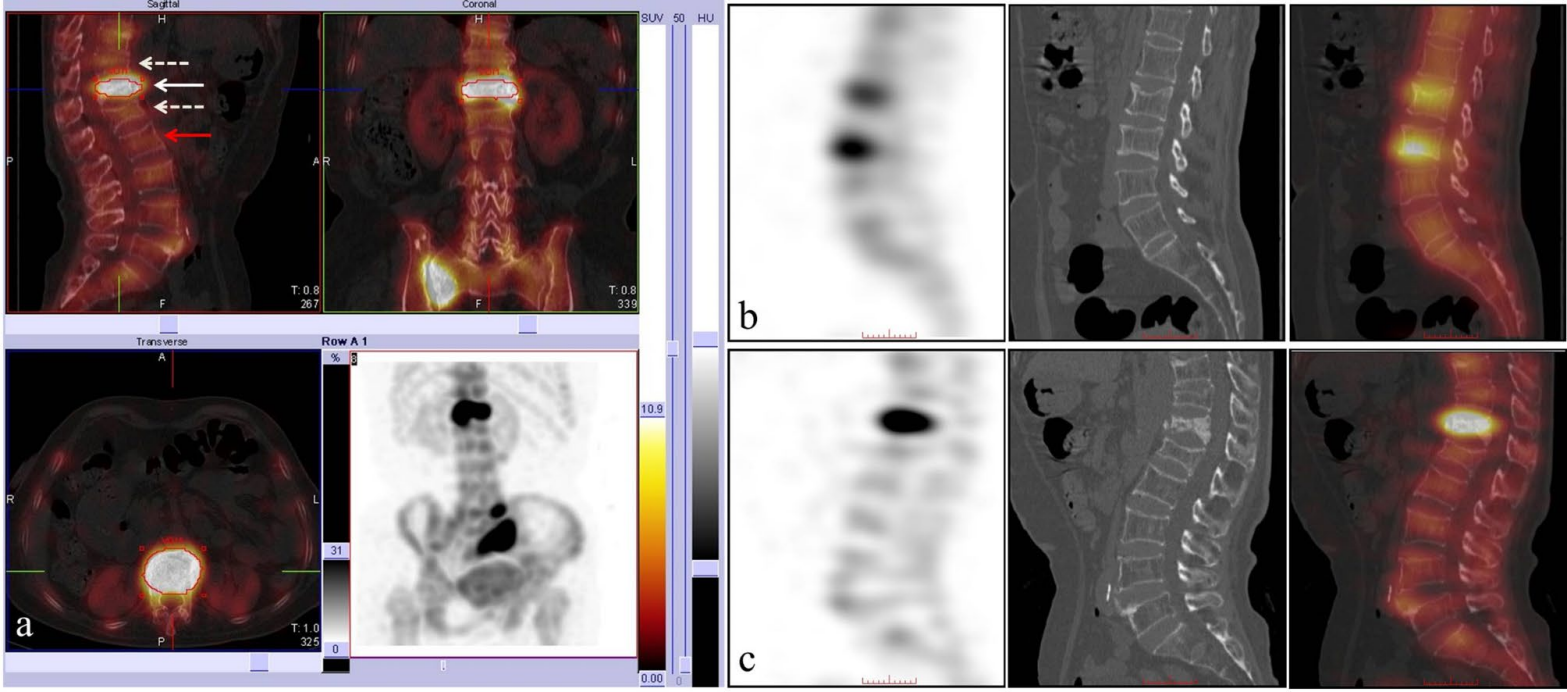
(**a**) The concentrated VOI of the vertebrae and the SUVmax (solid white arrow) were automatically drawn by the software. For normal vertebrae in the fresh compression fracture group, the line demarcates the cancellous region of the vertebrae body near the compression fracture (dotted white arrow). If the nearby vertebra body was an old VCF, the next adjacent vertebra was selected to outline (solid red arrow). (**b**) and (**c**) Fresh VCFs were regrouped based on the site of fracture. The sagittal SPECT, sagittal CT, and sagittal SPECT/CT of fresh VCFs are shown from left to right. Image **b** shows the concentration of ^99m^Tc-MDP located in the marginal sites. Image **c** shows the concentration of ^99m^Tc-MDP located in the central region of the vertebrae. VOI = volume of interest, SUVmax = the maximum standard uptake value, VCF = vertebral compression fracture

We used a nuclear medicine software package for quantitative analysis of SPECT/CT imaging. The obtained voxel-based volume activities were converted to SUVs using the xSPECT Quant application, accounting for patients’ weight, the injected activity, the residual activity in the syringe after administration and the time between injection and acquisition. Three-dimensional volumes of interest were placed over the positive lesion and over the adjacent normal vertebral spongiosa (Fig. 2a). SUVmax and CT values of the lesions and normal vertebrae were recorded. In addition, the vertebral bodies in control groups were randomly selected, and the SUVmax and CT values were recorded.

### Statistical analysis

Statistical analyses were conducted using R software version 4.0.3. Count data were described as frequency (percentage). Fisher’s exact probability method was used for the statistical analysis of count data. In the presence of normal distribution, measurement data were described as mean (standard deviation), and *t*-test or ANOVA was used for statistical analysis. In the absence of normal distribution, data were described as median [25% spacing number, 75% spacing number]. For statistical analysis, either the Games-Howell test or Mann-Whitney test was used. The test level α was set at 0.05. The receiver operator characteristic (ROC) curve was used to determine the optimal critical value of SUVmax.

## Results

### Patients

Of all 34 patients included in this study, 14 were males and 20 were females. Their median age was 72 years (range: 49–88) and their median pain course (the day of pain until the examination day) was 19 days (range: 3 days to 70 days). Fifteen patients had no obvious trauma, and the remaining had a clear history of trauma, such as severe cough, falls, and lifting heavy things. The characteristics of patients are summarized in Table 1. One patient was treated conservatively for multiple fractures, five patients received conservative treatment for other reasons, and the remaining 28 patients were offered cement augmentation. The pain was relieved after the surgery. Preoperative bone scintigraphy was conducted for all patients. Twenty-three patients underwent MRI, which showed the presence of vertebral edema, and the remaining patients underwent X-ray or CT scan. Among the 34 patients, a single VCF occurred in 18 patients, and more than one VCF occurred in 16 patients.

**Table 1:** Baseline characteristics of patients included in the study

Indicies (unit)	Normal range		*N*
Age (years)		72.0 [68.5;78.0]	34
Sex, *n* (%)			
Female		20 (58.8%)	
Male		14 (41.2%)	
P (mmol/L)	0.85–1.51	1.13 [1.06;1.27]	34
Ca (mmol/L)	2.11–2.52	2.21 [2.14;2.34]	34
Symptoms duration (Days)		19.0 [14.0;59.0]	34

Data are represented as median **[**interquartile range**].** P, phosphorus; Ca, calcium

### Detection results and characteristics of bone scan and SPECT/CT imaging

**Fig. 3:**
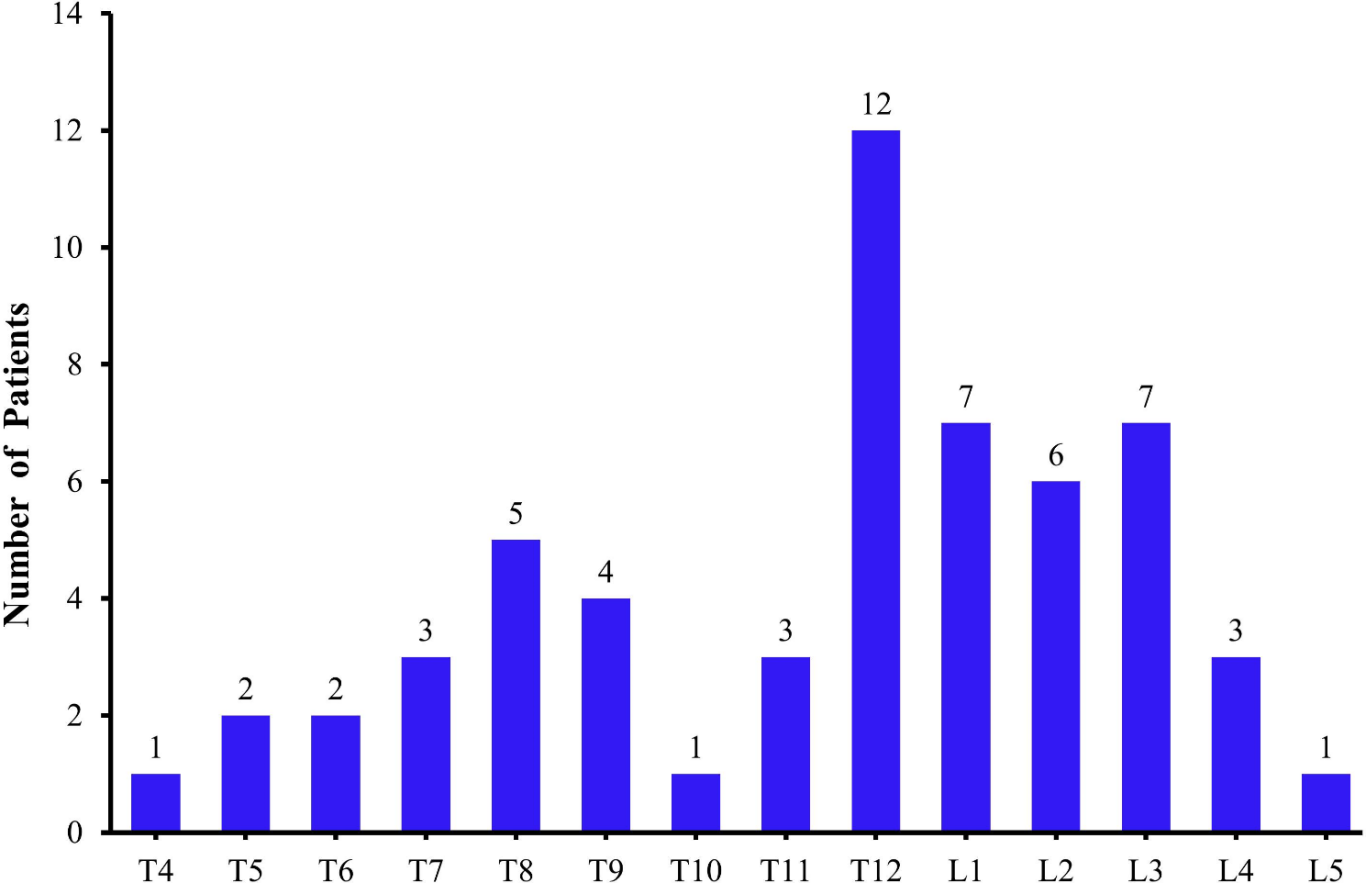
Distribution of fresh vertebral compression fracture

In total, 57 positive lesions were detected in 34 patients on the [^99m^Tc]-MDP scan. The number of fracture segments is shown in Fig. 3. Radiotracer uptake markedly increased in the linear pattern throughout all fresh VCF bodies in sagittal SPECT/CT imaging. Based on the location of [^99m^Tc]-MDP within vertebra, two types were defined: intervertebral type and marginal type (Fig. 2bc). Twenty of 57 lesions belonged to the intervertebral type. They were located in the intervertebral body linear uptake of [^99m^Tc]-MDP in the vertebral cancellous area, accompanied by biconcave or crush fracture in SPECT/CT imaging. Other 37 lesions belonged to the marginal type. They were located in the vertebral margin with linear uptake of [^99m^Tc]-MDP in the vertebral marginal area, accompanied by wedge deformation and increased bone density in SPECT/CT imaging. Among the vertebral marginal sites, 22 were in the superior margin of the vertebral body, and the remaining 15 were located in the inferior margin of the vertebral body.

In addition, 19 old VCFs in patients and 10 old VCFs in the control group were found in SPECT/CT imaging.

### Semi-quantitative analysis of [99mTc]-MDP SPECT/CT imaging

**Fig. 4:**
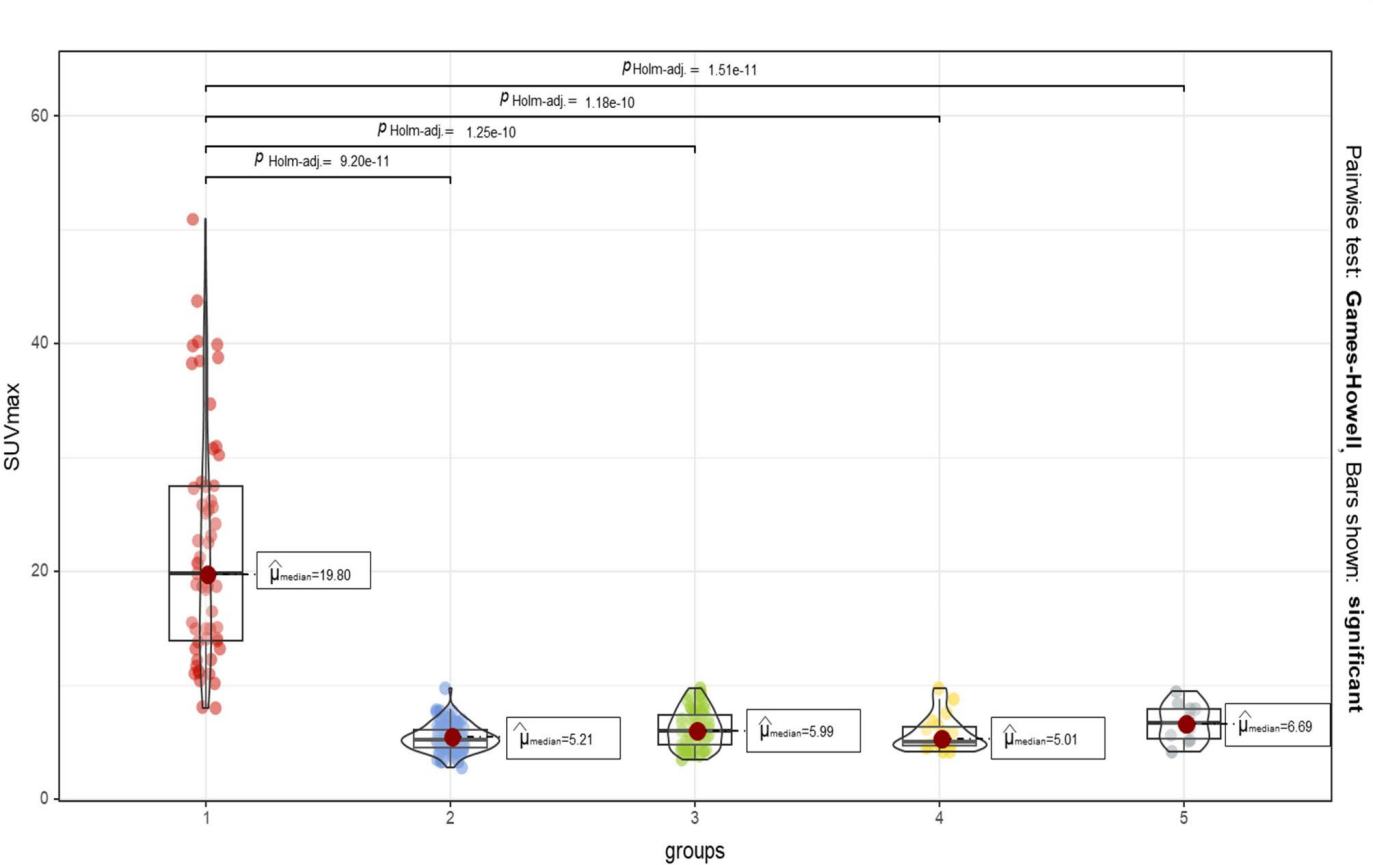
The violin diagram shows the median SUVmax values of the five groups. The median SUVmax of fresh VCFs was 19.8, which was significantly higher than that of the other four groups. SUVmax = the maximum standard uptake value

**Fig. 5:**
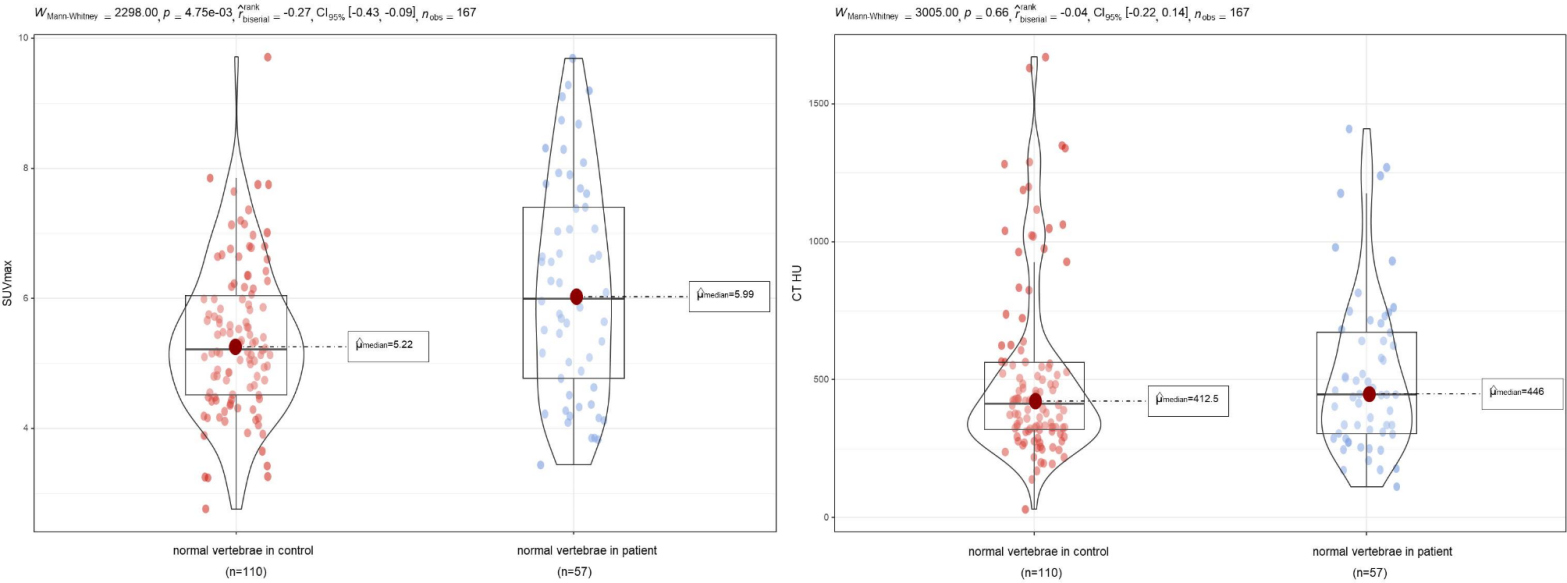
The comparison of SUVmax and CT HU values of normal vertebrae between the control group and the patient group. The median SUVmax, but not CT HU value, of normal vertebrae was significantly higher in the patient group than in the control group. CT = computed tomography. HU = Hounsfield units. CI = confidence interval

In total, 253 vertebrae were outlined. Among them, there were 57 fresh VCFs of vertebral bodies in the patient group (Group 1), 57 vertebral bodies adjacent to the fresh VCFs of vertebral bodies (Group 3), 110 normal vertebral bodies in the control group (Group 2), 19 old VCFs of vertebral bodies in the patient group (Group 4), and 10 old VCFs of vertebral bodies in the control group (Group 5). The median SUVmax of Group 1 was significantly higher than that of other groups (Fig. 4). The ROC curve showed that the cut-off value of SUVmax in diagnosing fresh VCF was 9.925, with the area under the curve of 0.998. In addition, the SUVmax in Group 3 was significantly higher than in Group 2 (*P*<0.001). Meanwhile, their CT values were not significantly different (Fig. 5). The difference between Group 4 and Group 5 was not statistically significant.

**Fig. 6:**
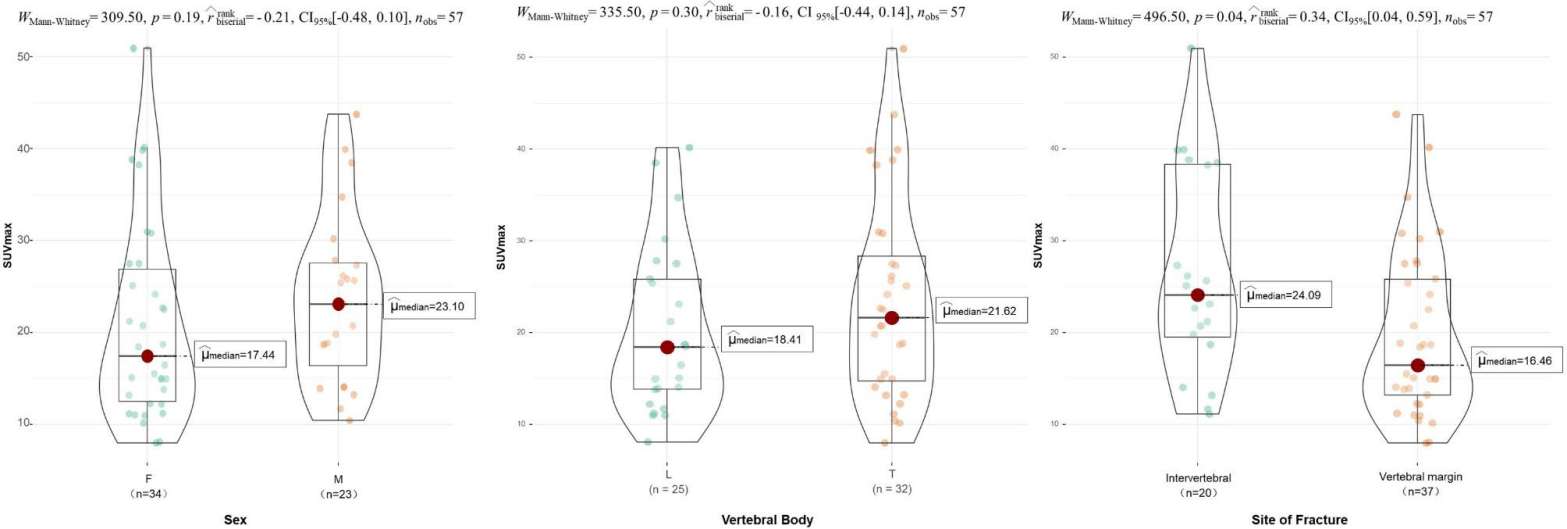
The median of SUVmax in subgroups of sex, vertebral body, and fracture site. The difference between intervertebral and vertebral margins was significant, but the difference between the other two subgroups was not significant. SUVmax = the maximum standard uptake value, CI = confidence interval, F = female, M = male, L = lumbar vertebra, T = thoracic vertebra

Figure 6 shows the median SUVmax of fresh VCF lesions regrouped by sex, vertebral body (thoracic and lumbar vertebrae), and fracture site. The SUVmax in the intervertebral site was significantly higher than that in the marginal site (*P* = 0.04), and no significant difference was observed between the other two subgroups.

## Discussion

Osteoporosis affects more than 200 million people worldwide. Accordingly, it is estimated that 30-50% of women and 20-30% of men will suffer from VCF [13], with a peak at 75–79 years [14]. Before treating the VCF, it must be determined whether it is a fresh or old fracture [15]. Bone imaging reflects the changes in blood flow and metabolism of vertebral bodies. Increased activity of radiopharmaceuticals can be observed in the fracture site [16]. Some studies proved the value of bone scan in diagnosing fresh VCFs [6–8, 11, 17], but most studies were qualitative. Our study adopted a quantitative analysis method, and ROC showed that the cutoff value of SUVmax for diagnosing fresh VCF in SPECT/CT was 9.925. Another study [17] also adopted quantitative technology and showed that the cutoff value of SUVmax for diagnosing fresh VCF was 18.5, which was higher than that found in our study. The different disease course of included patients may be the cause of the SUVmax discrepancy. Given that the SUVmax of the normal vertebral body and old VCFs in that study were also higher than those found in our study, we speculate the discrepancy may mostly originate from differences in analysis software and techniques. Despite differences in resources, both studies demonstrated that the SUVmax in fresh VCFs was significantly higher than that in normal vertebrae and old VCFs. Continuous multiple vertebral fractures (MVCFs) or discontinuous MVCFs are frequently caused by high-energy trauma and mild trauma, respectively [18]. It is advised to perform balloon kyphoplasty (BKP) at the most accumulated vertebrae on bone scintigraphy to achieve good clinical results in patients with MVCFs [9]. Compared with visual assessments for the most concentrated level of VCF on bone scintigraphy, the application of SUVs in bone scan can accelerate the diagnosis and help select the most accumulated level of fresh VCF more accurately.

One of the clinical indicators of surgical management by multivariate logistic regression analysis was osteoporotic fracture classification [19]. VCF is classified based on the morphological changes in vertebral height, and the severity of fracture increases in higher grades of this classification. However, this classification sometimes may be inaccurate, especially in distinguishing the grade 1 of VCF from spinal deformity [13]. The classification criteria are not suitable for hunchback patients [20]. In addition, other scholars described the shape of vertebral fractures as wedge, biconcave, and crushed using the semi-quantitative method, which is based on the degree of bone deformity (mild, moderate and severe) [21]. According to the vertebral fracture site, we classified the VCF sites into intervertebral and marginal types, which respectively correspond to the biconcave or crushed type and wedge type in the previous report [21]. This suggests that intervertebral type with higher SUVmax was more severe than marginal type. Moreover, the degree of VCF in thoracolumbar osteoporotic fracture was strongly and positively correlated with the cancellous bone CT Hounsfield units (HU) value [22]. On the contrary, the CT HU value of the adjacent vertebral body was not significantly different between the intervertebral group and the marginal group in this study. Therefore, quantitative analysis of bone imaging can be used to classify the severity of VCFs and may be more objective than traditional anatomical measures based on vertebral height changes or bone density.

Osteoporotic fractures are prone to multiple VCFs, which often occur in adjacent vertebral bodies, especially in the elderly population [23]. Meanwhile, previous reports showed the incidence of vertebral re-fracture is high one year after surgery [22, 24], mostly occurring in the adjacent vertebrae [25]. VCF is currently characterized by macroscopic structural destruction of the vertebral body [26]. The review [13] considered microscopic trabecular fracture and repair, vertebral endplate/cortex fracture without vertebral deformity, and vertebral wedge/crush fracture as presentations of compromised vertebral bone. Our results showed that the SUVmax of the normal vertebral body was significantly higher in the patient group before the surgery than in the control group. It is believed that the new fracture after cement augmentation of VCF is caused by the shift of the normal load transmission through the weakened osteoporotic spine [27]. As the normal vertebrae were adjacent to the vertebrae with a compression fracture in this study, the adjacent vertebrae showed microstructural changes due to the first trauma or the shifting load. Microscopic changes altered vertebral height after the secondary trauma. These microscopic changes were not reflected in CT HU values due to the non-significant differences in this study. Some studies investigated the risk factors of vertebral re-fracture [24, 25, 28]. Our results showed that quantitative bone scintigraphy may be useful in predicting vertebral re-collapse.

VCF is usually caused by low-energy trauma in elderly patients with osteoporosis [15]. The risk of elderly fracture increases with the decrease in bone mineral density (BMD) [29]. BMD can reflect the degree of osteoporosis and is the best indicator for assessing the risk of osteoporotic fracture [30]. Currently, clinicians adopt dual-energy X-ray absorptiometry (DXA) to measure BMD, and osteoporosis is confirmed when T-score is ≤-2.5 [29]. However, DXA has important limitations [31]. Recently, a study used CT HU value as an indicator of BMD for diagnosing osteoporosis [32] and predicting the risk of re-fracture after percutaneous kyphoplasty (PKP) [33]. MRI was also used for quantitative assessment of osteoporotic bones [31]. Hydroxyapatite is the main mineral component of bone [29] and has a higher similarity to the true composition of the mineral component of bone than calcium [10]. As one of the bisphosphonates, [^99m^Tc]-MDP used for bone imaging is mainly absorbed on the surface of hydroxyapatite. In the future, we will conduct large-size prospective studies to investigate whether the SUV value of bone imaging can be used for diagnosing osteoporosis or predicting fracture risk in osteoporotic patients.

We acknowledge some limitations to this study. Firstly, our study was limited by the relatively small sample sizes; thus, we could not perform further analyses. Secondly, there was no follow-up to confirm the clinical value of the difference between the SUVmax of normal vertebrae in patients and the control group, as this study was a retrospective study. Thirdly, due to the small amount of data, most patients did not undergo DXA examination, and we did not analyze the relationship between DXA and SUVmax. Therefore, larger sample sizes and prospective studies are needed in the future.

## Conclusion

The quantitative technique of bone SPECT/CT can provide the concentration of radiopharmaceutical in the fractured vertebral body, which has a significant value in diagnosing fresh VCF. It can also distinguish the severity of the fracture and is especially helpful for those with concomitant vertebral deformity. In addition, it needs further investigation whether the higher SUVs of the vertebrae adjacent to the fractured vertebra suggest the possibility of microstructural changes and a higher risk of future re-fracture.

## Data Availability

The datasets generated and/or analysed during the current study are available from the corresponding author on reasonable request.

## Abbreviations

[^99m^Tc]-MDP: Technetium-99 m-methyldiphosphonate
BKP: Balloon kyphoplasty
BMD: Bone mineral density
CT: Computed tomography
DXA: Dual X-ray absorptiometry
HU: Hounsfield units
MRI: Magnetic resonance imaging
MVCFs: Multiple vertebral fractures
PKP: Percutaneous kyphoplasty
ROC: Receiver operator characteristic
SPECT/CT: Single-photon emission computed tomography / computed tomography
SUV: Standard uptake value
VCF: Vertebral compression fractures
